# Slow and rapid auxin responses in Arabidopsis

**DOI:** 10.1093/jxb/erae246

**Published:** 2024-05-25

**Authors:** Zilin Zhang, Huihuang Chen, Shuaiying Peng, Huibin Han

**Affiliations:** College of Bioscience and Bioengineering, Jiangxi Agricultural University, Jiangxi, Nanchang, 330045, China; Institute of Science and Technology Austria (ISTA), 3400 Klosterneuburg, Austria; College of Bioscience and Bioengineering, Jiangxi Agricultural University, Jiangxi, Nanchang, 330045, China; College of Bioscience and Bioengineering, Jiangxi Agricultural University, Jiangxi, Nanchang, 330045, China; University of Warwick, UK

**Keywords:** Auxin, ABP1, ABLs, slow and rapid responses, TIR1/AFBs, TMKs

**The TIR1/AFB–Aux/IAA–ARF canonical auxin signaling pathway is widely accepted to (de)active transcriptional regulation, thus controlling auxin-associated developmental processes. However, the theme of a rapid auxin response has emerged since the 2018 Auxins and Cytokinin in Plant Development conference. To date, a few signaling components have been identified to mediate both slow and rapid auxin responses, which unveils the complexity of auxin signaling.**

## The nuclear TIR1/AFB2–AFB5 receptors mediate the transcription-dependent slow auxin response

The plant phytohormone auxin coordinates cellular responses to various developmental and environmental signals, thus optimizing plant growth and development. Auxin controls not only the slow transcriptional responses, but also transcriptional-independent rapid responses occurring in seconds (Table 1; Box 1; Das *et al*., 2021; Dubey *et al*., 2021; Fiedler and Friml, 2023). Genetic screens using an auxin-regulated sustained root growth assay have led to the identification of signaling components that are involved in slow transcriptional regulations including the TRANSPORT INHIBITOR RESPONSE1/AUXIN-SIGNALING F-BOX (TIR1/AFB) receptors (Dharmasiri *et al*., 2005; Kepinski and Leyser, 2005), AUXIN/INDOLE-3-ACETIC ACID (Aux/IAA) co-repressors (Gray *et al*., 2001; Kepinski and Leyser, 2004), and the AUXIN RESPONSE FACTOR (ARF) transcription factors (Tiwari *et al*., 2003; Weijers *et al*., 2005). In general, when the cellular auxin level is low, Aux/IAA repressors bind to ARFs via the PB1 domain and prevent ARF-driven gene transcription. At high cellular auxin concentrations, auxin enters into the TIR1/AFB2–AFB5-binding pocket to initiate ubiquitination and proteasomal degradation of Aux/IAA proteins, thus releasing the transcriptional activities of ARFs (Das *et al*., 2021). Notably, ETTIN/ARF3 directly binds to auxin and determines transcriptional regulation of auxin signaling independent the TIR1/AFB receptors (Simonini *et al*., 2016). The nuclear auxin signaling pathway can explain the majority of auxin effects on plant growth. However, several developmental processes including rapid root growth inhibition (Fendrych *et al*., 2018; Li *et al*., 2021), Ca^2+^ influxes (Shih *et al*., 2015), cytoplasmic streaming (Friml *et al*., 2022), apoplast alkalinization (Li *et al*., 2021), and membrane depolarization (Serre *et al*., 2021) are too fast to be mediated by TIR1/AFB2–AFB5-dependent transcriptional regulation, suggesting that a rapid auxin-responsive system exists.

**Table 1.** A summary of TIR1/AFB and ABP1/ABL/TMK receptors in slow and rapid auxin responses

Response type	Receptors	Signaling events	Developmental processes	References
Slow responses	TIR1	The E3 ligase activity of TIR1 is required for Aux/IAA degradation	Root growth	Kepinski and Leyser (2004) Yu *et al.* (2013)
		Production of cAMP via AC activity of TIR1	Root growth and gravitropic response	Qi *et al.* (2022)
		E12K substitution in TIR1 causes defective SCF complex assembly	Root and shoot growth	Yu *et al.* (2015)
		D170E and M473L mutations in TIR1 increase Aux/IAA protein degradation	Root and shoot development	Yu *et al.* (2013)
		Regulation of SAUR19 transcription and H+-ATPase activity via TIR1/AFB receptors	Hypocotyl growth	Fendrych *et al.* (2016)
		RALF1 peptide activates TIR1/AFB pathway-dependent transcriptional regulation	Root growth	Li *et al*. (2022)
		Production of cGMP via GC activity of TIR1	Unknown	Qi *et al.* (2023)
	TMK1	The C-terminal kinase domain of TMK1 phosphorylates IAA32/34, and the phosphorylated IAA32/34 protein inhibits its WAV3-dependent ubiquitination	Apical hook development	Cao *et al.* (2019); Wang *et al.* (2024)
	TMK4	TMK4 regulates TAA1 function	Root development	Wang *et al.* (2020)
	ABP1/TMK1	Fast protein phosphorylation	Auxin canalization	Friml *et al.* (2022)
	TMK1	TMK1 phosphorylates AHA2	Hypocotyl elongation	Lin *et al.* (2021)
	TMK1	Phosphorylation of MPKK4/5 and MPK3/6 by TMK1/TMK4	Lateral root formation	Huang *et al.* (2019)
	TMK1	MAKR2 negatively regulates TMK1 function and PIN2 asymmetry distribution	Root gravitropic response	Marquès-Bueno *et al*. (2021)
	TMK1/TMK4	TMK1 and TMK4 phosphorylate auxin transporter PIN1	Shoot development	Wang *et al.* (2022)
	TMK1	TMK1 phosphorylates auxin transporter PIN2	Root gravitropic response	Rodriguez *et al.* (2022)
	ABL1/ABL2	Unknown	Hypocotyl growth, pavement cell development, leaf morphology	Yu *et al.* (2023)
Rapid responses	AFB1	Fast membrane depolarization	Fast root growth inhibition	Serre *et al.* (2021)
	AFB1	AUX1, AFB1, and CNCG14 determine Ca2+ flow and pH gradient at the root transition zone	Fast root growth inhibition	Serre *et al.* (2023); Qi *et al.* (2023)
	AFB1	Production of cGMP (not sure about the involvement of AFB1)	Fast root growth inhibition	Qi *et al.* (2023)
	ABP1/AFB1/TMK1	Fast protein phosphorylation	Unknown	Friml *et al.* (2022); Kuhn *et al*. (2024)
	ABP1/ABL1/ABL2	Fast protein phosphorylation	Unknown	Yu *et al.* (2023)
	TMK1/ABP1/ABL1 ABL2	Fast phosphorylation of RAF-kinases	Cytoplasmic streaming	Kuhn *et al*. (2024)
	TMK1/TIR1–AFBs	TMK1 and TIR1/AFB receptors antagonistically regulate AHA activity and H+ flow	Fast root growth inhibition	Li *et al*. (2021)

## Cytoplasmic localized AFB1 is specifically involved in rapid auxin response

The six TIR1/AFB proteins play overlapping and specialized roles in auxin signaling (Prigge *et al*., 2020). It has been suggested that the transcriptional-dependent growth responses are mainly mediated by nuclear-localized TIR1/AFB2–AFB5 receptors, while cytoplasmic-localized AFB1 is specifically required for rapid membrane depolarization and auxin-dependent inhibition of root growth (Prigge *et al*., 2020; Serre *et al*., 2021; Chen *et al*., 2023; Dubey *et al*., 2023). When AFB1 localizes to the nucleus, it cannot rescue the auxin-induced sustained root growth inhibition of the *tir1 afb2* mutant, implying that nuclear-localized AFB1 could not functionally replace TIR1 for its transcriptional regulation, probably due to its inability to form the E3 ubiquitin SCF complex (Chen *et al*., 2023). Additionally, nuclear-localized AFB1 could no longer mediate the rapid auxin effect on root growth inhibition. Conversely, cytosol-localized TIR1 could not rescue the rapid root growth inhibition of the *afb1* mutant (Chen *et al*., 2023). Furthermore, auxin does not induce cytosolic Ca^2+^ influx and rapid membrane depolarization in the *afb1* mutant roots (Serre *et al*., 2021; Dubey *et al*., 2023). Taken together, cytoplasmic AFB1 is the major receptor mediating rapid, non-transcriptional responses, and it cannot replace the transcriptional activity of TIR1 (Box 1).

## cAMP is responsible for slow transcriptional auxin responses while cGMP accounts for rapid auxin responses

cAMP is an important second messenger in mammalian signaling pathways; it also functions in plant signaling cascades (Qi and Friml, 2023). The adenylate cyclase (AC) motif is identified and conserved in the TIR1/AFB receptors from early-diverging land plants, suggesting that TIR1/AFB receptors could utilize ATP to produce cAMP in plants (Qi *et al*., 2022). Indeed, exogenous application of auxin stimulates AC activity and increases the cAMP level in roots. Mutation in the AC domain specifically abolishes the AC activity and severely compromises TIR1 function in mediating sustained root growth inhibition and auxin-induced transcription (Qi *et al*., 2022). Notably, AC activity of TIR1 is not required for the rapid auxin responses, including Ca^2+^ transients, apoplastic alkalinization, and fast root growth inhibition (Qi *et al*., 2022), implying that AC activity is crucial for TIR1-dependent transcriptional regulation. Nonetheless, how the AC activity of AFB1 contributes to the rapid auxin response remains to be determined. Whether cAMP regulates TIR1 ubiquitin ligase activity, Aux/IAA degradation, or ARF transcriptional activity also remains elusive.

cGMP is another well-known second messenger, produced from GTP by guanylate cyclase (GC) (Qi and Friml, 2023). A guanylate cyclase (GC) motif is also identified in the TIR1/AFB receptors but only in angiosperm species (Qi *et al*., 2023, Preprint). GC activity and the cGMP level are rapidly induced at 1 min after auxin treatment. The supplementation of cell-permeable dibutyryl-cGMP, an analog of cGMP, causes a similar CNGC14-dependent Ca^2+^ spike and auxin-induced fast root growth inhibition. However, no genetic evidence supports that GC mutation in AFB1 is unable to produce cGMP and is also unresponsive to auxin (Qi *et al*., 2023, Preprint). Collectively, TIR1/AFB2–AFB5-dependent cAMP regulates the slow transcriptional auxin responses, while further genetic evidence needs to be provided to confirm the AFB1-depedent cGMP or cAMP production for the rapid and non-transcriptional responses (Box 1) (Qi *et al*., 2022, 2023, Preprint). Whether cGMP or cAMP impacts rapid auxin-induced phosphorylation remains to be addressed (Friml *et al*., 2022). Moreover, the physiological significance of the dynamic balance between the cGMP and cAMP level triggered by auxin still requires further investigation. We also cannot exclude the possibility that other players can stimulate cAMP and cGMP production to regulate slow and rapid auxin responses.

## The ABP1–ABL1/2–TMK cell surface receptor complex is required for the auxin-induced slow and rapid responses

Cell surface-localized auxin receptors have been proposed for years, and AUXIN BINDING PROTEIN 1 (ABP1) and TRANSMEMBRANE KINASEs (TMKs) are good candidates (Napier, 2021). ABP1 and TMKs bind to the natural auxin indole-3-acetic acid (IAA) and the synthetic auxin 1-naphthaleneacetic acid (NAA). The *abp1* mutant exhibits a defect in auxin canalization (Friml *et al*., 2022), and the *tmk1* mutant displays multiple auxin-associated developmental defects such as lateral root formation, hypocotyl growth, and apical hook development (Dai *et al*., 2013; Xu *et al*., 2014; Cao *et al*., 2019; Huang *et al*., 2019; Lin *et al*., 2021). Notably, TMK1 plays antagonistic roles with TIR1/AFB receptors that converge on rapid root growth inhibition, while ABP1 is not involved (Li *et al*., 2021).

Auxin triggers a specific and ultrafast protein phosphorylation in Arabidopsis roots within 2 min independent of the TIR1/AFB receptors (Friml *et al*., 2022; Kuhn *et al*., 2024). ABP1 and TMK1 also contribute to auxin-induced fast protein phosphorylation (Friml *et al*., 2022). When comparing the rapid phospho-response in wild-type Arabidopsis roots with that of *tmk1* and *abp1* mutants, the auxin-induced hyperphosphorylation response is almost completely abolished and overlapping hypophosphorylation sites are also found between *tmk1* and *abp1* mutants, suggesting that TMK1 and ABP1 act together in mediating an auxin-triggered rapid phospho-response (Friml *et al*., 2022). Additionally, TMK activity is not activated in the *abp1* mutant. Notably, *abp1* and *tmk1* mutants are defective in long-term canalization (Friml *et al*., 2022). These results suggest that ABP1 is required for the auxin-triggered activation of TMK signaling, and both components are required for a subset of rapid and slow cellular auxin responses (Friml *et al*., 2022).

Recently, ABP1-LIKE PROTEIN 1 (ABL1) and ABL2, belonging to the GERMIN-LIKE PROTEIN (GLP) family, have been identified via an immunoprecipitation–mass spectrome analysis, and they are believed to form a cell surface complex with ABP1 and TMKs to sense auxin (Yu *et al*., 2023). ABL1 and ABL2 are localized to the apoplast, and they interact with TMKs and can directly bind to auxin (Yu *et al*., 2023). The *abl1 abl2* double mutant displays morphological defects such as pavement cell shape, curling leaves, and reduced seedling size. Furthermore, auxin-induced fast protein phosphorylation is abolished in the *abp1 abl1 abl2* triple mutant. However, it is not clear whether there is an overlap of phosphorylated proteins between *abp1* and *abp1 abl1 abl2* mutants (Friml *et al*., 2022; Yu *et al*., 2023). Additionally, the *abp1 abl1 abl2* mutant phenotype seems to be unstable and would be affected by unknown environmental variables or other GLPs (Yu *et al*., 2023; Kuhn and Weijers, 2024). Taken together, ABP1–ABLs–TMKs form a cell surface complex to regulate both slow and rapid auxin response, but this probably requires further examination.

## RAF-kinases mediate rapid auxin-induced protein phosphorylation

Auxin induces a fast protein phosphorylation, but which kinase can relay this rapid phosphorylation remains unclear (Friml *et al*., 2022). Recently, the RAPIDLY ACCELERATED FIBROSARCOMA (RAF)-LIKE KINASE (RAF) subfamily, belonging to the mitogen-acivated protein kinase kinase kinase (MAPKKK) family, is identified as mediating the rapid auxin-triggered phospho-response (Kuhn *et al*., 2024). The phosphorylation level of RAFs in the *abp1*, *tmk1*, and *abp1 abl1 abl2* mutants is remarkably disrupted, but not in the *afb1* mutant, indicating the cell surface-based auxin perception for rapid activation of RAFs (Friml *et al*., 2022; Yu *et al*., 2023; Kuhn *et al*., 2024). Unexpectedly, auxin still induces a fast root growth inhibition and membrane depolarization in *raf* mutants, but RAFs are indispensable for auxin-triggered fast cytoplasmic streaming (Kuhn *et al*., 2024). Furthermore, RNA-sequencing analysis shows no obvious auxin-triggered transcriptional changes between the wild type and *raf* mutants. Taken together, RAFs play an essential and distinct role in mediating auxin-triggered rapid responses, but not in transcriptional regulation. Auxin induces the C-terminal cleavage of the TMK1 kinase domain to phosphorylate IAA32 and IAA34 (Box 1; Cao *et al*., 2019), whether this C-terminus of TMK1 can activate RAFs needs to be verified. It would also be appealing to uncover the unidentified kinases and substrates that can endow the rapidity of RAF action.

## Future perspectives

Arabidopsis have developed multiple signaling pathways to control both slow and rapid auxin-triggered responses (Box 1); both TIR1/AFB and ABP1–ABs–TMK pathways seems to uncouple slow responses from rapid responses (Lin *et al*., 2021; Friml *et al*., 2022; Yu *et al*., 2023; Qi *et al*., 2022, 2023). Despite their role in the rapid auxin response, TIR1/AFB and ABP1–TMK receptors are also associated with long-term canalization defects (Mazur *et al*., 2020; Friml *et al*., 2022). On the other hand, rapid phosphorylation of AHA2 by TMK1 leads to sustained slow hypocotyl growth (Fendrych *et al*., 2016; Lin *et al*., 2021). The unanswered question is how the slow and rapid auxin responses are integrated by developmental and environmental cues via the distinct nuclear and cell surface auxin signaling pathways. Do Ca^2+^, cAMP/cGMP, and H^+^-ATPases (AHAs) act as ‘gatekeepers’ for the slow and rapid auxin response, because they are rapidly induced by auxin, but contribute to the slow response? Also, it is unclear how the rapid phosphorylation changes triggered by AFB1 and ABP1/ABL–TMK complexes contribute to the integration of slow and rapid auxin responses.

Auxin extends far beyond Arabidopsis and is found in all land plants and algae (Carrillo-Carrasco *et al*., 2023), while the nuclear auxin response pathway seems to be restricted to land plants, as charophytes lack TIR1/AFB receptors (Blázquez *et al*., 2020). TMKs are deeply conserved in plants, while ABP1 and ABL1/2 are ancient and extend well beyond land plants, indicating that the ABP1–ABL–-TMK module is ancient and generic (Carrillo-Carrasco *et al*., 2023). The identification of auxin-induced rapidly phosphorylated proteins in bryophytes and algae (Kuhn *et al*., 2024) would provide more information about the physiological significance of auxin-induced rapid responses in bryophyte and algal development, and will also provide novel insights into the evolution of TIR1/AFB and ABP1–ABL–TMK pathways.

Box 1. **Multiple roads to slow and rapid auxin responses in Arabidopsis**

(A) The AUX1 auxin influx transporter and passive diffusion deliver auxin into the cells, then intracellular auxin is mainly perceived by nuclear-localized TIR1/AFB2–AFB5 receptors to trigger a rapid CNGC14-mediated Ca^2+^ influx. The Ca^2+^ transient contributes to the H^+^ influx into cells across the plasma membrane (PM) via so far unknown H^+^ carriers, which ultimately leads to apoplast alkalinization, PM depolarization, and fast root growth inhibition. On the other hand, TMK1 perceives the extracellular auxin signal to phosphorylate AHA2, resulting in apoplast acidification and promotion of root growth. Hence, TIR1/AFBs and TMK1 play antagonistic roles in auxin-induced rapid root growth inhibition. (B) Auxin stimulates GC activity via the cytoplasmic-localized AFB1 receptor to produce the second messenger cGMP. cGMP then probably triggers a Ca^2+^ influx through the CNGC14 channel to induce H^+^ influx via unknown H^+^ carriers, resulting in rapid root growth inhibition. (C) Auxin triggers AC activity of TIR1/AFB receptors to generate cAMP; the cAMP then regulates auxin-triggered slow root growth inhibition. (D) Auxin binds to the nuclear TIR1/AFB2–AFB5 receptors to initiate the degradation of Aux/IAA, thus releasing the transcriptional activity of ARFs and gene expression. (E) The cell surface receptor complex ABP1–ABL1/ABL2–TMK recognizes extracellular auxin signal to phosphorylate and activate RAF-kinases to trigger rapid protein phosphorylation and auxin-associated cytoplasm steaming. (F) Auxin induces the cleavage of the TMK1 C-terminal kinase domain; the cytoplasmic TMK1 kinase domain is translocated to the nucleus to phosphorylate and stabilize IAA32 and IAA34 transcription repressors. Phosphorylated IAA32 and IAA34 inhibit WAV3-dependent ubiquitination of IAA32 and IAA34 to regulate apical hook development. (G) Auxin binds to TMK1 and TMK4 and phosphorylates MKK4/5 and MPK3/6 to control lateral root development. (H) TMK4 phosphorylates TAA1, an auxin biosynthesis protein, to control root development. Abbreviations: AHA2, H^+^-ATPase 2; ARF, AUXIN RESPONSE FACTOR; CNGC14, CYCLIC NUCLEOTIDE-GATED CHANNEL 14; TIR1/AFB, TRANSPORT INHIBITOR RESPONSE1/AUXIN-SIGNALING F-BOX PROTEIN; TMK1, TRANSMEMBRANE KINASE 1; AUX1, AUXIN RESISTANT 1; ABL1, ABP1-LIKE PROTEIN 1; ABL2, ABP1-LIKE PROTEIN 2; ABP1, AUXIN BINDING PROTEIN 1; KD, kinase domain; P, phosphorylation; Ub, ubiquitination. A dashed line indicates no direct evidence.

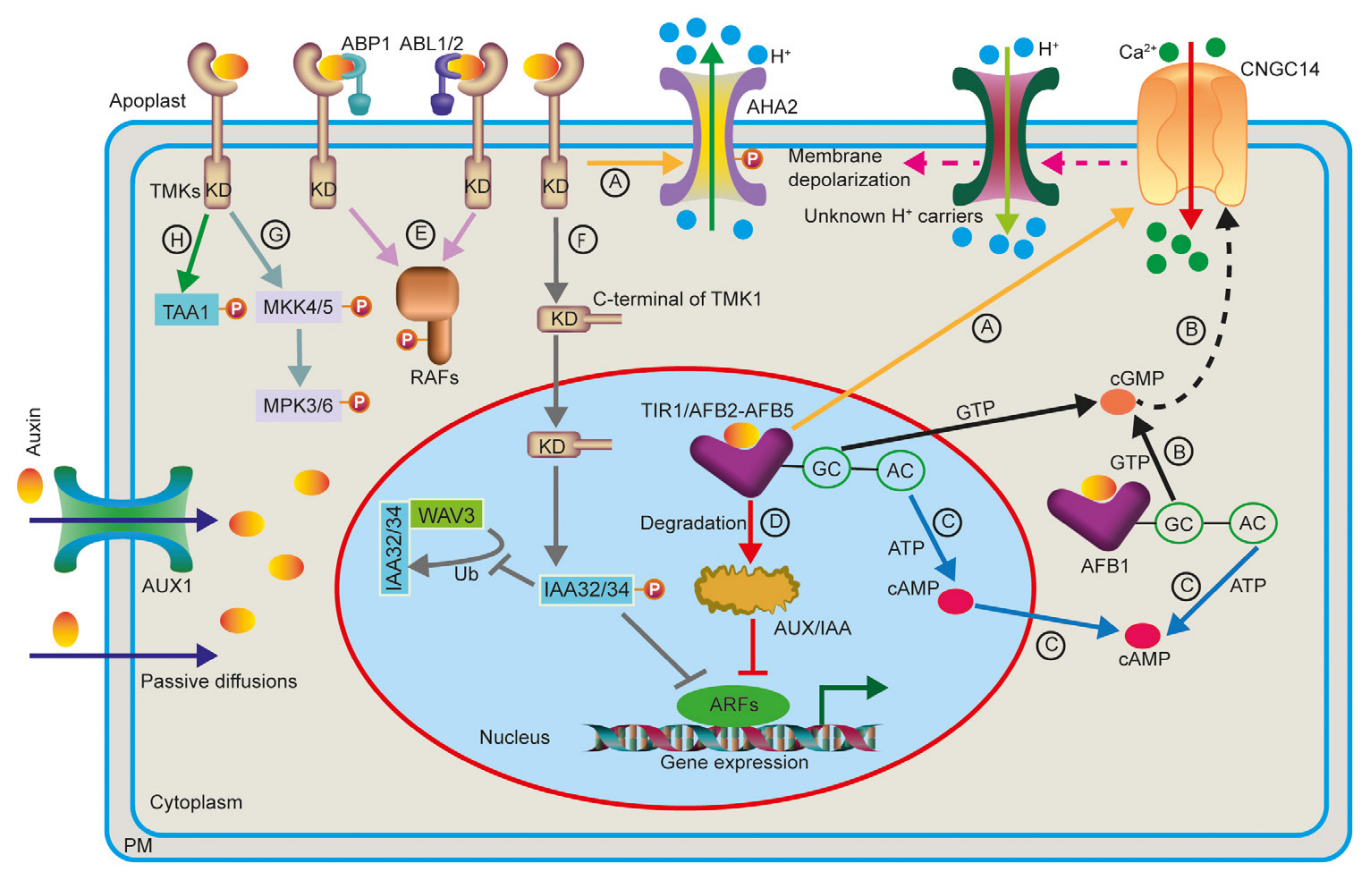

## Data Availability

No new data were generated in this article.
